# Tau pathology is associated with postsynaptic metabotropic glutamate receptor 5 (mGluR5) in early Alzheimer's disease in a sex‐specific manner

**DOI:** 10.1002/alz.70004

**Published:** 2025-02-25

**Authors:** Yan Wang, Jie Wang, Xing Chen, Zengping Lin, Zhiwen You, Kun He, Tengfei Guo, Jun Zhao, Qi Huang, Ruiqing Ni, Yihui Guan, Binyin Li, Fang Xie

**Affiliations:** ^1^ Department of Nuclear Medicine & PET Center, Huashan Hospital Fudan University Shanghai China; ^2^ Department of Nuclear Medicine, Shanghai East Hospital Tongji University School of Medicine Shanghai China; ^3^ Central Research Institute United Imaging Health Care Group Co., Ltd. Shanghai China; ^4^ Institute of Biomedical Engineering Shenzhen Bay Laboratory Shenzhen China; ^5^ Institute for Biomedical Engineering, Institute for Regenerative Medicine University of Zurich & ETH Zurich Zurich Switzerland; ^6^ Department of Neurology and Institute of Neurology, Ruijin Hospital Shanghai Jiao Tong University School of Medicine Shanghai China

**Keywords:** Alzheimer's disease, metabotropic glutamate receptor 5 (mGluR5), synapse, tau

## Abstract

**INTRODUCTION:**

To investigate the associations of metabotropic glutamate receptor 5 (mGluR5) with tau deposition and cognitive ability in patients with early Alzheimer's disease (AD).

**METHODS:**

Twenty‐six cognitively impaired (CI) and 14 cognitively unimpaired (CU) individuals underwent mGluR5 positron emission tomography (PET) ([^18^F]PSS232), amyloid PET ([^18^F]florbetapir), and tau PET ([^18^F]MK6240), and neuropsychological assessment. The relationships among mGluR5 availability, tau deposition, and neuropsychological assessment were analyzed using Spearman's correlation and mediation analyses.

**RESULTS:**

CI patients had lower mGluR5 in the hippocampus than CU (standardized uptake value ratio [SUVr]: 2.03 ± 0.25 vs 1.79 ± 0.17, *p* = 0.003). Hippocampal mGluR5 was negatively associated with hippocampal tau deposition (*r* = −.46, *p* = 0.003) and positively associated with cognitive performance, but only in women. Hippocampal tau deposition mediated the effect of mGluR5 on cognitive performance.

**DISCUSSION:**

Reduced hippocampal mGluR5 is negatively related with tau deposition in most cortical regions and positively associated with cognitive performance, making it a promising biomarker for AD diagnosis and therapy.

**Highlights:**

- Cognitively impaired (CI) patients exhibited lower metabotropic glutamate receptor 5 (mGluR5) availability in the hippocampus than cognitively unimpaired (CU) subjects.
- Hippocampal mGluR5 availability was negatively associated with tau deposition in widespread cortex.
- Hippocampal mGluR5 availability was positively associated with cognitive performance.
- The close association of mGluR5 with tau and cognition performance exists only in females.
- Tau pathology mediated the relationship between mGluR5 availability and cognition.

## 1 BACKGROUND

Alzheimer's disease (AD) is a progressive neurodegenerative disorder characterized by extracellular amyloid plaques and intracellular tau neurofibrillary tangles (NFTs). However, significant progress in understanding AD pathogenesis has broadened our understanding of its neuropathological changes. Synaptic loss is considered one of the major pathologies and has been associated with cognitive impairment in AD and mild cognitive impairment (MCI).^1^, ^2^ Proteomic studies of cerebrospinal fluid (CSF) from patients with preclinical AD have revealed changes in synapse protein levels that precede neurodegeneration, suggesting that synapse pathology is one of the hallmarks of AD.^3^

Metabotropic glutamate receptor 5 (mGluR5) primarily modulates synaptic transmission and plasticity by activating G protein–regulated signal transduction mechanisms.^4^, ^5^, ^6^ Aberrant mGluR5 signaling and related synaptic failure play crucial roles in the initial pathophysiological mechanism of AD.^7^ Considerable evidence suggests that mGluR5 acts as a receptor for potentially hyperphosphorylated tau and amyloid beta (Aβ)42, and transduces their signals into synapses, causing synaptic failure.^8^, ^9^, ^10^, ^11^, ^12^ Moreover, tauopathy in AD may be triggered by the Aβ42‐mediated activation of mGluR5. Tau is found mainly in axons, but in AD brains, hyperphosphorylated, misfolded, and oligomeric tau can be found in both pre‐ and postsynaptic terminals.^13^, ^14^, ^15^ Studies in tau‐transgenic mice have shown that increased tau levels at the synaptic terminal can lead to synaptic loss and dysfunction.^16^, ^17^, ^18^, ^19^ However, these interlinked pathological pathways were found in mouse models, and further research is needed to understand how tau interacts with the postsynaptic system in humans.

mGluR5 also serves as a postsynaptic biomarker because it is located mainly at the postsynapse and is also found in astrocytes.^20^ Recently, positron emission tomography (PET) radiotracers have been utilized to monitor alterations in mGluR5 expression levels under physiological and pathological conditions with high selectivity and specificity. Previous studies have revealed significant reductions in mGluR5 within the hippocampus in individuals with MCI due to AD or mild AD dementia via [^18^F]3‐Fluoro‐5‐[(pyridin‐3‐yl)ethynyl]benzonitrile ([^18^F]FPEB) and [^18^F]PSS232).^21^, ^22^, ^23^ Furthermore, the mGluR5‐silent allosteric modulator (SAM) Bristol‐Myers Squibb‐984923 (BMS‐984923)6‐(fluoro‐^18^F)‐3‐(1H‐pyrrolo[2,3‐c]pyridin‐1‐yl)isoquinolin‐5‐amine can completely restore synaptic density in aged mouse models of AD, and the therapeutic benefit persists even after drug washout. It can also reduce phospho‐tau accumulation in double knock‐in mice.^24^ These results revealed the significant role of mGluR5 in tau phosphorylation.

Our previous work revealed that tau deposition–induced presynaptic loss and tau pathology were also closely associated with the presynaptic biomarker of synaptic vesicle glycoprotein 2A (SV2A), as assessed via PET.^25^ We also observed that presynaptic density, as determined by SV2A via PET, is closely associated with postsynaptic mGluR5.^26^ However, the associations of mGluR5 with the neuropathological hallmarks of AD, especially with tau pathology, remain unclear. Due to the involvement of mGluR5 in AD pathology and its therapeutic potential, investigating the correlations among various biomarkers in the complex pathogenesis of AD is highly intriguing to elucidate the development and progression of the disease. In the present study, we sought to explore the spatial assessment of mGluR5 distribution and tau deposition in cognitively impaired (CI) and cognitively unimpaired (CU) individuals via PET scans using [^18^F]PSS232 and 6‐(fluoro‐^18^F)‐3‐(1H‐pyrrolo[2,3‐c]pyridin‐1‐yl)isoquinolin‐5‐amine ([^18^F]MK6240) while exploring the associations of mGluR5 with tau deposition and their associations with AD biomarkers and cognitions.

## 2 METHODS

### 2.1 Participants

Forty participants from the memory clinic and communities in Shanghai, including 26 CI individuals (18 AD and 8 MCI) and 14 CU individuals, were recruited for this single‐center, cross‐sectional study. All participants underwent PET/MRI (magnetic resonance imaging) scans with [^18^F]PSS232 to investigate mGluR5 and PET/CT (computed tomography) scans with [^18^F]florbetapir and [^18^F]MK6240 to detect Aβ and tau deposition. All examinations were completed within 1 month. This study was approved by the Institutional Ethical Review Board of Huashan Hospital, Fudan University, and Ruijin Hospital affiliated with Shanghai Jiao Tong University. Written informed consent was obtained from the participants.

AD was identified according to the 2018 National Institute on Aging and Alzheimer's Association (NIA‐AA) diagnostic criteria, and MCI was diagnosed based on Jak and Bondi's criteria after clinically diagnosed AD was excluded, as we reported previously.^27^ MCI patients or early AD patients (MMSE ≥ 18) with amyloid‐positive were included in this study.^23^ Patients without cognitive impairment who were Aβ negative were classified as CU. Participants with a history of conditions that may affect brain structure or function (e.g., stroke or any neurodegenerative diseases), severe diseases (e.g., cancer), or alcohol or drug abuse were excluded. Participants with a history of smoking were also excluded.^28^

### 2.2 Neuropsychological assessment

All participants underwent a series of revised neuropsychological tests designed for the Chinese population, as described in our previous study.^29^, ^30^, ^31^ The neuropsychological tests included the animal fluency test ([AFT], total score) and Boston Naming Test ([BNT], 30 items) for the language domain, Parts A and B (time to completion) of the shape trials test (STT) for the executive function domain, and the Auditory Verbal learning Test long time delayed free‐recall (AVLT‐LDR) and the delayed recognition scores (24 items, AVLT‐REC) for the episodic memory domain. The Montreal Cognitive Assessment‐Basic (MoCA‐B)^32^ and Mini‐Mental State Examination (MMSE) were used to evaluate global cognition.

### 2.3 Image acquisition

A 30‐min static PET scan started at 30‐min post‐injection of [^18^F]PSS232 (injected activity ∼185 MBq) using a 3T PET/MR scanner (uPMR790, United Imaging Health Care, Shanghai, China). T1‐weighted MR images were acquired simultaneously to obtain whole‐brain anatomic volumetric images (repetition time = 7200 ms, echo time = 3.0 ms, flip angle = 10°, acquisition matrix = 256 × 329, in‐plane resolution = 1 mm × 1 mm, slice thickness = 1 mm, slices = 176). The 3D Dixon images were used for attenuation correction. The static PET data were reconstructed using all list mode events by ordered subset‐expectation maximization algorithm (20 subsets and 4 iterations) with the following parameters: field of view = 300 × 300 mm^2^, matrix size = 150 × 150, voxel size = 2.0 × 2.0 × 2.0 mm^3^. The data were reconstructed after correction for randomness, dead time, scatter, and attenuation.

The 20‐min static [^18^F]florbetapir scans were conducted 50 min after injection (injected activity ∼370 MBq), and the 20‐min static [^18^F] MK6240 scans were obtained 90–110 min after injection (injected activity ∼185 MBq) using a PET/CT scanner (Biograph Truepoint HD 64 PET/CT, Siemens, Erlangen, Germany). The acquired PET data were reconstructed using a filtered back‐projection algorithm and subsequently corrected for decay, normalization, dead time, attenuation, scatter, and random coincidences.

### 2.4 Data preprocessing

The Statistical Parametric Mapping 12 (SPM12) toolbox in MATLAB R‐2018b (Welcome Trust Centre for Neuroimaging, London, UK; https://www.fil.ion.ucl.ac.uk/spm) was employed for image preprocessing following a previously described procedure.^33^, ^34^, ^35^, ^36^ The steps are as follows: (1) The PET and T1‐weighted MR images are reoriented, and the PET images are coregistered to the individual T1‐weighted images; (2) the T1 images were segmented into gray matter (GM), white matter (WM), and CSF and then warped into the standard Montreal Neurologic Institute (MNI) space simultaneously with the coregistered PET images; and (3) a Gaussian smoothing kernel with an 8‐mm full width at half maximum (FWHM) is used to smooth the images. For the [^18^F]MK6240 PET data, we masked the meninges using GM segmentation to minimize the interference of meningeal spillover into adjacent brain regions.^37^

**RESEARCH IN CONTEXT**

1. **Systematic review**: Metabotropic glutamate receptor 5 (mGluR5) primarily modulates synaptic transmission and plasticity. Aberrant mGluR5 signaling and related synaptic failure play crucial roles in the initial pathophysiological mechanism of Alzheimer's disease (AD). Tauopathy in AD may be triggered by the amyloid beta (Aβ)42‐mediated activation of mGluR5. We searched PubMed, and we found no literature reporting the association of mGluR5 availability with synaptic density.
2. **Interpretation**: In this study, we found that cognitively impaired patients exhibited lower mGluR5 availability in the hippocampus. mGluR5 was negatively associated with tau deposition and positively associated with cognitive performance only in women. Tau deposition mediated the effect of mGluR5 on cognitive performance.
3. **Future directions**: The future research direction is to observe the longitudinal changes in mGluR5 availability and tau burden, and to investigate how tau deposition affects mGluR5 expression. This will further our understanding of the relationship between mGluR5 expression and tau deposition during the progression of AD and its underlying mechanisms.

Standardized uptake value ratio (SUVr) was calculated for [^18^F]PSS232 and [^18^F]MK6240 using the pons and inferior cerebellar as the reference region, respectively.^23^ For [^18^F]MK6240 and [^18^F]florbetapir PET, a visual reading was performed by three specialists in nuclear medicine and was dichotomously interpreted as positive or negative in this study. Hippocampal volume was segmented by CAT12 and corrected for individual intracranial volume (formula: hippocampal/intracranial volume × 1000). This value was calculated as the hippocampal volume ratio (HpVr).

For visual reading for PET images, three board‐certified nuclear medicine physicians, including at least one senior physician, were included to read these images. And they were all blinded to the clinical, demographic, and neurological information. Amyloid PET was visually determined as in our previous report.^30^ The interpretation of tau PET adhered to the guidelines: Readers directed their attention to eight predetermined brain regions in each hemisphere of the cerebral cortex (16 regions in total): hippocampus, mesial temporal, inferior temporal, lateral temporal, parietal, posterior cingulate, occipital, and frontal lobes. In assessing each of the 16 cortical regions, the reader determined whether there was an abnormally elevated radiotracer presence in the region compared to the cerebellum.^38^, ^39^, ^40^, ^41^ The results were recorded if more than two physicians made the same judgment and were then divided into tau PET positive (T+) and negative (T−) groups.

For quantification of tau deposition, Braak stages were defined based on a previous PET study using the Desikan–Killiany–Tourville atlas as follows: Braak I (entorhinal cortex), Braak II (hippocampus), Braak III (amygdala, parahippocampal gyrus, fusiform gyrus, and lingual gyrus), Braak IV (insular, inferior temporal, posterior cingulate, and inferior parietal cortices), Braak V (orbitofrontal, superior temporal, inferior frontal, cuneus, anterior cingulate, supramarginal gyrus, lateral occipital, precuneus, superior parietal, superior frontal, and rostromedial frontal cortices), and Braak VI (paracentral, postcentral, precentral, and pericalcarine gyri).^42^, ^43^

### 2.5 Postmortem human brain tissue and multiplex immunofluorescence staining

Postmortem hippocampal brain tissue from five patients with AD, each with a clinical diagnosis confirmed by pathological examination, and five CU subjects were included in this study (detailed information in Table S1). The brain tissues were obtained from the Netherlands Brain Bank (NBB), the Netherlands, with all materials collected from donors or from whom written informed consent was obtained for a brain autopsy, and the materials and clinical information for research purposes were obtained by the NBB. The study was conducted according to the principles of the Declaration of Helsinki and subsequent revisions. All the postmortem human brain tissue experiments were carried out in accordance with ethical permission obtained from the regional human ethics committee and the medical ethics committee of the VU Medical Center for NBB tissue. Multiple immunofluorescences and immunohistochemical stains were performed on 3‐µm‐thick slices from AD patients and CU subjects. Microscopy and quantification of fluorescence intensity were performed as described previously.^26^ The primary and secondary antibodies used are listed in Table S2 and included rabbit anti‐mGluR5 (1:500), mouse anti‐Aβ1‐16, 6E10 (1:1000), and mouse anti‐p‐tau, AT8 (1:500) antibodies.

### 2.6 Statistical analysis

Group comparisons were performed via the Wilcoxon signed‐rank test or independent samples *t*‐test for continuous variable intergroup comparisons depending on the data distribution determined via the Kolmogorov‒Smirnov normality test and via chi‐square analysis for categorical variables. The intergroup differences in demographic data, neuropsychological scores, and voxel of interest (VOI)‐based PET SUVr values were assessed with a significance threshold of *p* < 0.05 (sex, age, and education years were adjusted as covariables). Voxelwise comparisons were also performed for whole‐brain PET SUVr, with the cluster‐defining voxel threshold set to the default value of 0.001 (sex, age, and education years were adjusted as covariates), *k*_ext_ = 100 voxels. The associations of regional mGluR5 with regional tau deposition and their correlations with cognitive outcome were assessed via Spearman's correlation analysis.

IBM SPSS 26.0 software (SPSS; Chicago, Illinois, USA) and GraphPad Prism 9.5 (GraphPad Software; La Jolla, California, USA) were used for the VOI‐based analysis, and the SPM12 toolbox was employed for the voxelwise analysis. To investigate whether the association between aberrant mGluR5 in the hippocampus and cognitive levels was regulated by tau deposition, we performed a mediation analysis via PROCESS v4.1. The significance of the mediation was assessed by calculating bias‐corrected 95% confidence intervals (CIs) by bootstrapping (5000 resamples).

## 3 RESULTS

### 3.1 Demographic characteristics and clinical assessments

The demographic information is given in Table 1. CI individuals displayed worse global cognition and memory, language, and executive cognition than CU individuals. Significant hippocampal atrophy was observed (HpVr: 3.49 ± 0.54 vs 4.00 ± 0.40, *p* = 0.004) in CI individuals compared with that in CU individuals. Furthermore, sex, age, or education level did not differ significantly between CI and CU individuals.

**TABLE 1:** Demographic characteristics and clinical assessments.

	Cognitive unimpairment (*n* = 14)	Cognitive impairment (*n* = 26)	*p* value
Women	6	17	0.198
Age, y	70.04 ± 7.28	69.77 ± 9.42	0.834
Education, y	13.86 ± 3.66	12.50 ± 3.94	0.474
MMSE score (0–30)	28.14 ± 1.41	22.04 ± 2.34	< 0.001
MoCA‐B	24.36 ± 1.15	16.31 ± 3.27	< 0.001
AVLT			
AVLT‐LDR	5.93 ± 1.64	0.62 ± 1.17	< 0.001
AVLT‐REC	21.36 ± 1.82	14.42 ±3.00	< 0.001
BNT score (0–30)	23.50 ± 2.96	18.54 ± 4.57	0.001
AFT	15.50 ± 4.35	10.73 ± 4.53	0.003
STTA	63.21 ± 26.10	116.00 ± 62.37	< 0.001
STTB	144.86 ± 56.73	221.62 ± 68.94	0.001
HpVr	4.00 ± 0.40	3.49 ± 0.54	0.004
*APOE* ɛ4 copy number (*n*)			0.001
2 copies	0 (0%)	2 (7.7%)	
1 copy	4 (28.6%)	20 (76.9%)	
0 copies	10 (71.4%)	4 (15.4%)	

*Note*: Values are presented as the means ± SDs when appropriate.

Abbreviations: AFT, animal fluency test; AVLT‐LDR, Auditory Verbal learning Test long time delayed free‐recall; AVLT‐REC, Auditory Verbal Learning Test recognition scores (24 items); BNT, Boston Naming Test; HpVr, hippocampal volume ratio; MMSE, Mini‐Mental State Examination; MoCA‐B, Montreal Cognitive Assessment‐Basic; STTA, shape trials test Part A; STTB, shape trials Part B.

### 3.2 Group differences in mGluR5 and tau deposition

**FIGURE 1 alz70004-fig-0001:**
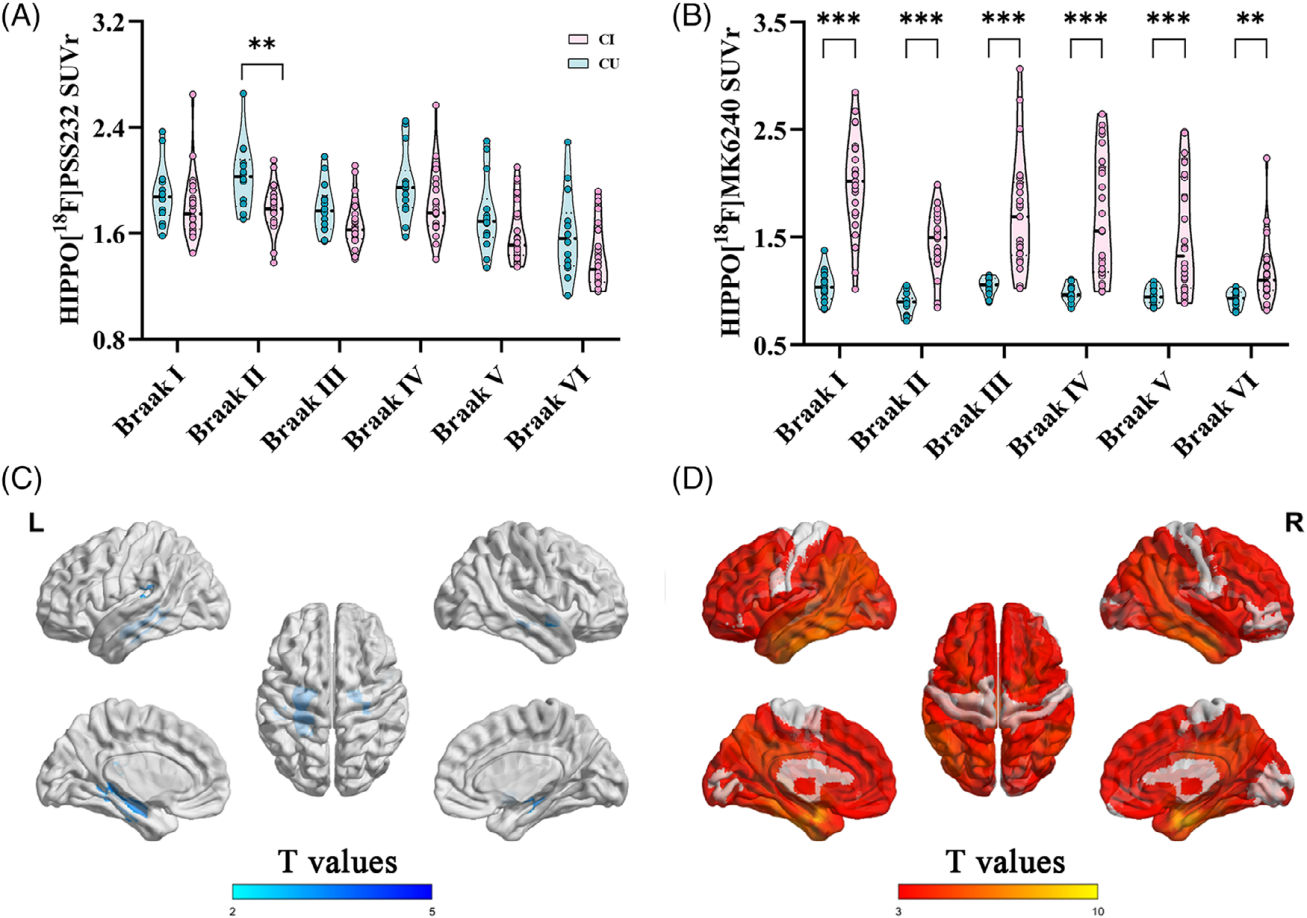
Distribution of decreased [^18^F]PSS232 (A, C) and increased [^18^F]MK6240 (B, D) in individuals with CI compared with those with CU according to the VOI and voxelwise analysis (sex, age, and education years were adjusted as covariates). Group comparisons between patients with CI and CU of mean SUVr values in different brain regions were performed with a two‐tailed unpaired *t*‐test with a significance threshold of *p* < 0.05. *p < 0.05, **p < 0.01, ***p < 0.001. CI, cognitively impaired; CU, cognitively unimpaired; SUVr, standardized uptake value ratio; VOI, volume of interest.

VOI‐based analysis revealed a significant reduction in mGluR5 in the hippocampus, also known as Braak II (SUVr: 1.79 ± 0.17 vs 2.03 ± 0.25, *p* = 0.003) in CI individuals compared with CU individuals. Compared with that in the CU, the [^18^F] MK6240 SUVr was greater in the hippocampus (1.45 ± 0.30 vs 0.88 ± 0.10, *p* < 0.001) and the entire neocortex, as shown in Figure 1. Voxelwise analysis revealed reduced mGluR5 in the hippocampus and increased tau deposition in most neocortical regions.

**FIGURE 2:**
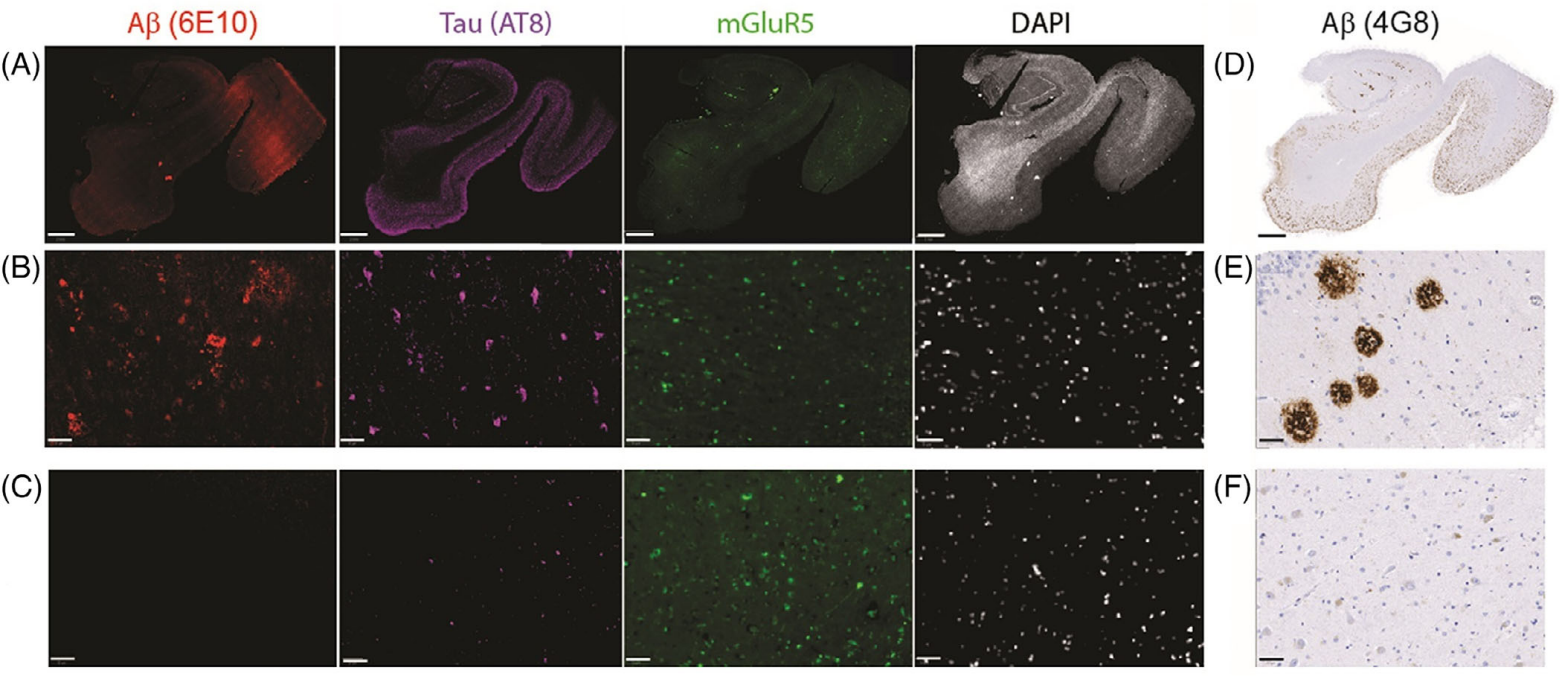
The levels of mGluR5 and Aβ and tau expression in the hippocampus in the AD patients were lower than those of individuals in the CU group. (A–C) The presence of diffuse and dense Aβ plaques (6E10 staining, red) and tau (AT8 staining, purple), with relatively lower fluorescence intensities of mGluR5 (green) in the hippocampus of AD patients compared with those in the CU group. (D–F) Representative 4G8 immunochemical staining for Aβ in hippocampal sections from HCs and AD patients. The nucleus was counterstained with DAPI (white). Scale bars = 2000 µm (A, B) and 50 µm (C). Aβ, amyloid beta; AD, Alzheimer's disease; AVLT‐REC, Auditory Verbal Learning Test delayed recognition scores (24 items); CU, cognitively unimpaired; DAPI, author to provide; HC, author to provide; mGluR5, metabotropic glutamate receptor 5; SUVr, standardized uptake value ratio.

Next, we assessed amyloid, tau, and mGluR5 expression in postmortem hippocampal slices from AD patients and CU subjects[Fig alz70004-fig-0001] (Figure 2). Compared with that in CU subjects, the expression of mGluR5 in the hippocampus was lower in AD patients (11.47 ± 3.10 vs 22.69 ± 4.24, *p* = 0.001). Immunofluorescence and immunochemical staining revealed 6E10‐ and 4G8‐positive Aβ plaques (both diffuse and dense) in the hippocampus of AD patients but rarely of CU subjects. The percentage of the 4G8 area covered by the hippocampus was greater in the AD patients than in the CU subjects (79.44 ± 7.51 vs 19.28 ± 6.60, *p* < 0.001). An increased average fluorescence intensity of phosphorylated tau (p‐tau) (AT‐8) was observed in the hippocampus of AD patients compared with CU subjects (10.15 ± 3.80 vs 18.36 ± 3.84, *p* = 0.009; Figure S1).

### 3.3 Associations of mGluR5 with tau deposition

**FIGURE 3:**
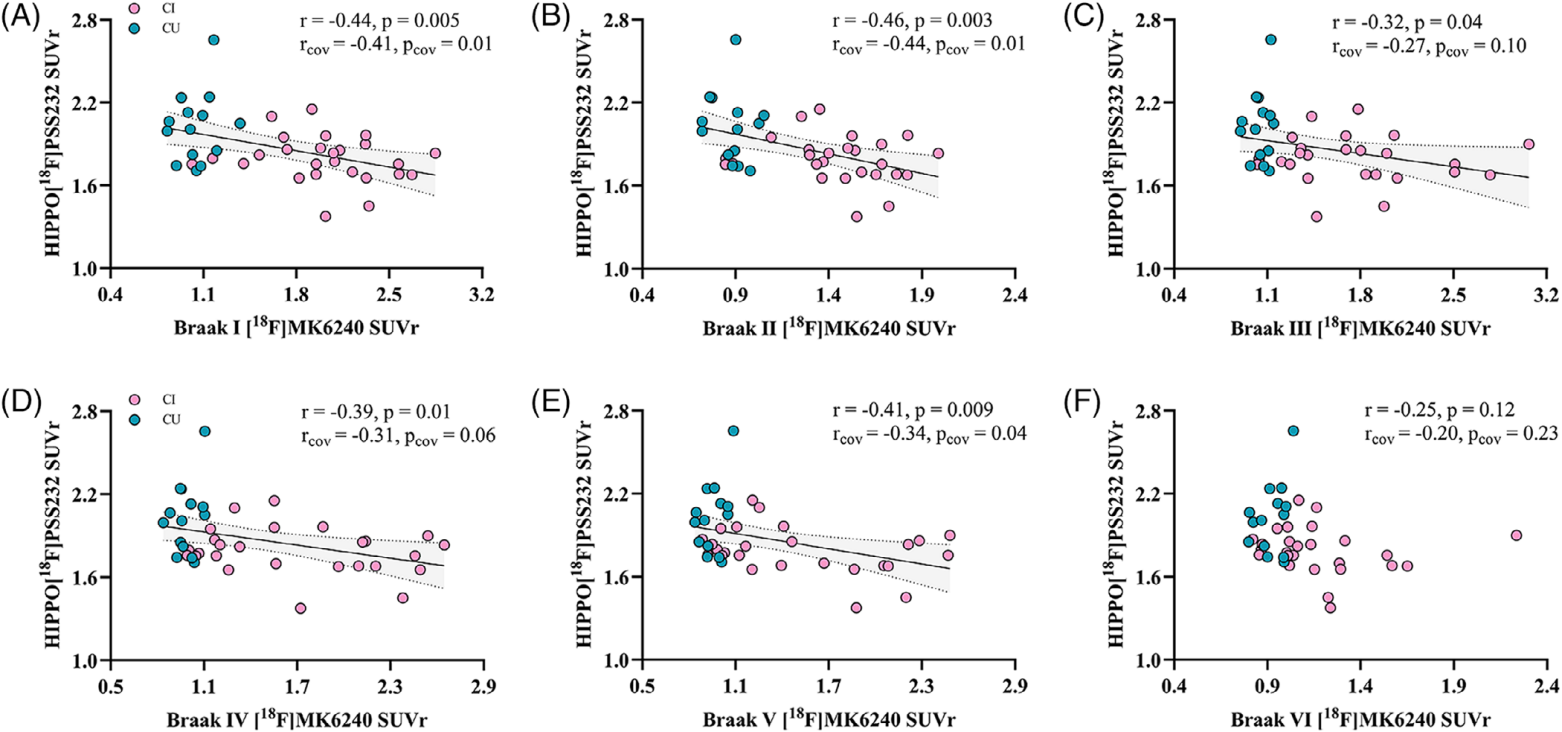
The correlation between the hippocampal [^18^F]PSS232 and [^18^F]MK6240 SUVr in the overall cohort. (A, C–F) Decreased hippocampal mGluR5 availability was associated with increased tau deposition in most of the neocortex. (B) Decreased hippocampal mGluR5 availability was associated with increased hippocampal tau deposition. HIPPO, hippocampus; mGluR5, metabotropic glutamate receptor 5; *p*_cov_, *p* value with sex, age, education years as a covariate; *r*_cov_, correlation coefficient with sex, age, education years as a covariate; SUVr, standardized uptake value ratio.

mGluR5 availability was negatively associated with tau deposition (*r* = −.39, *p* = 0.03) in the hippocampus (also known as Braak II), as shown in Figure 3. Moreover, hippocampal mGluR5 was also associated with tau deposition in Braak I (*r* = −.44, *p* = 0.005), Braak III (*r* = −.32, *p* = 0.04), Braak IV (*r* = −.39, *p* = 0.01), and Braak V (*r* = 0.41, *p* = 0.009). After adjusting for sex, age, and education years, the correlation remained significant in Braak stage I (*r*_cov_ = −.41, *p*_cov_ = 0.01), II hippocampus (*r*_cov_ = −.44, *p*_cov_ = 0.01), and V (*r*_cov_ = −.34, *p*_cov_ = 0.04) regions. However, hippocampal tau deposition was not significantly correlated with the [^18^F]PSS232 SUVr in most Braak stages except the Braak II (hippocampus, Figure S2). Furthermore, in the regional‐to‐regional association analysis, there was no significant correlation except in the Braak II region (Figure S3).

### 3.4 Associations between mGluR5 and neuropsychological assessments

**FIGURE 4:**
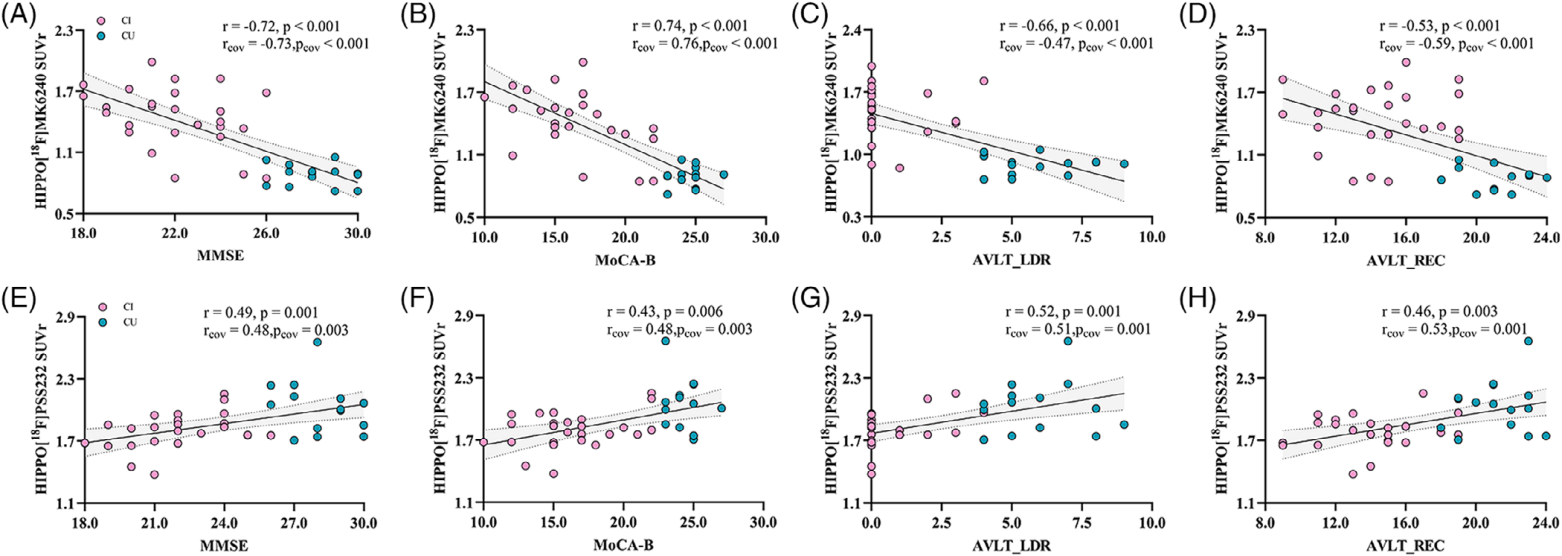
Correlations of the hippocampal [^18^F]MK6240 SUVr and [^18^F]PSS232 SUVr with different cognition scores. (A–D) Correlations of hippocampal [^18^F]MK6240 with the MMSE, MoCA‐B, AVLT‐LDR, and AVLT‐REC scores. (E–H) Correlations of hippocampal mGluR5 availability with the MMSE, MoCA‐B, AVLT‐LDR, and AVLT‐REC scores. AVLT‐LDR, author to provide; AVLT‐REC, Auditory Verbal Learning Test delayed recognition scores (24 items); mGluR5, metabotropic glutamate receptor 5; MMSE, Mini‐Mental State Examination; MoCA‐B, Montreal Cognitive Assessment‐Basic; SUVr, standardized uptake value ratio.

mGluR5 availability in the hippocampus was correlated with MMSE (*r* = 0.49, *p* = 0.001), MoCA‐B (*r* = 0.43, *p* = 0.006), AVLT‐LDR (*r* = 0.52, *p* = 0.001), and AVLT‐REC scores (*r* = 0.46, *p* = 0.003). The [^18^F]MK6240 SUVr in the hippocampus was also associated with the MMSE score (*r* = −.72, *p* < 0.001), MoCA‐B score (*r* = −.74, *p* < 0.001), AVLT‐LDR score (*r* = −.66, *p* < 0.001), and AVLT‐REC score (*r* = −.53, *p* < 0.001). These results remained significant after adjusting for sex, age, and education years, as shown in Figure 4.

### 3.5 Sex differences in the association of mGluR5 with tau and cognitive decline

**FIGURE 5:**
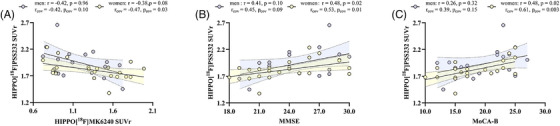
Correlations of the hippocampal [^18^F]PSS232 SUVr with [^18^F]MK6240 SUVr and different cognitions are only significant in women. A significant interaction of hippocampal mGluR5 was found in correlation with hippocampal tau deposition (A), MMSE (B), and MoCA‐B (C). mGluR5, metabotropic glutamate receptor 5; MMSE, Mini‐Mental State Examination; MoCA‐B, Montreal Cognitive Assessment‐Basic SUVr, standardized uptake value ratio.

We examined whether sex influenced the associations of hippocampal mGluR5 with tau and cognitive decline. We found that increased mGluR5 availability was correlated with lower tau deposition in the hippocampus in women (*r*_cov_ = −.38, *p*_cov_ = 0.03, Figure 5A). Consistently, we found decreased hippocampal mGluR5 was related to the MMSE (*r*_cov_ = 0.53, *p*_cov_ = 0.01; Figure 5B), and MoCA‐B (*r*_cov_ = 0.61, *p*_cov_ = 0.003; Figure 5C) in women.

### 3.6 The associations between mGluR5 and cognition are mediated by tau pathology

**FIGURE 6:**
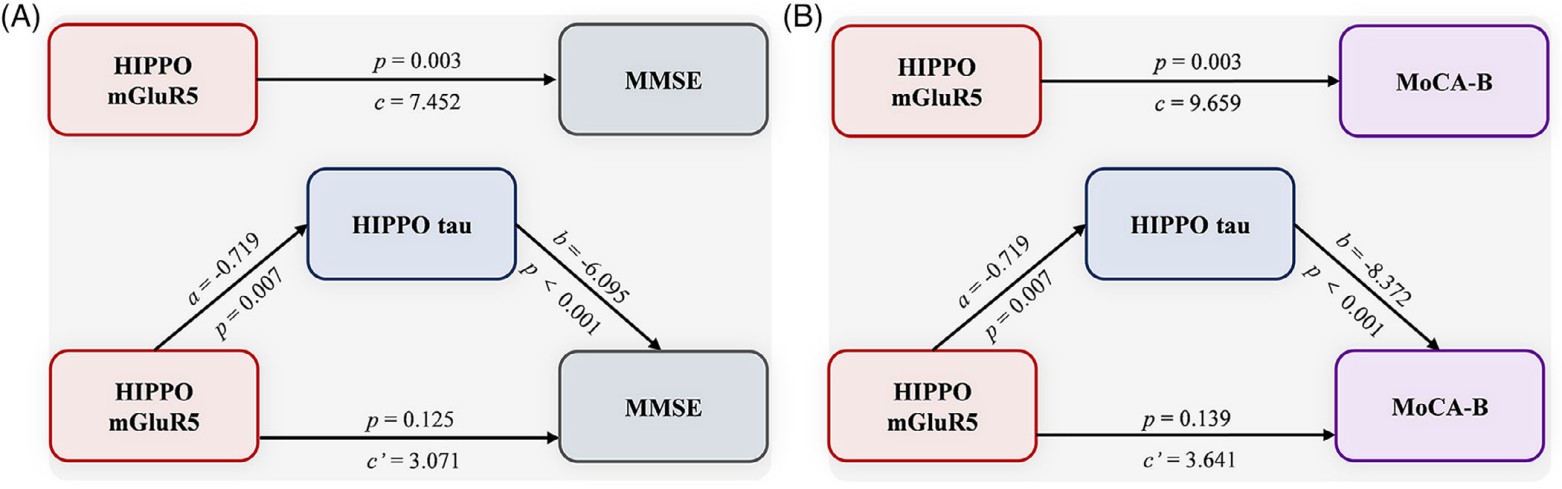
Analysis of the effects of tau pathology on the associations between hippocampal mGluR5 levels and cognitive decline (MMSE and MoCA‐B scores). (A) Tau pathology mediates the relationships between hippocampal mGluR5 levels and MMSE scores; (B) tau pathology mediates the relationships between hippocampal mGluR5 levels and MoCA‐B scores; sex, age, and education years were adjusted as covariates. mGluR5, metabotropic glutamate receptor 5; MMSE, Mini‐Mental Status Examination; MoCA‐B, Montreal Cognitive Assessment‐Basic.

We performed a mediation analysis to investigate the effect of hippocampal tau deposition on the association between mGluR5 availability and cognition. As shown in Figure 6, the associations of hippocampal mGluR5 with the MMSE and MoCA‐B scores were fully mediated by hippocampal tau deposition (MMSE score: indirect effects = 4.38, standard error [SE] = 1.67, 95% CIs = 1.74 to 8.39; direct effects = 3.07, SE = 1.95, 95% CIs = −0.89 to 7.03; MoCA‐B score: indirect effects = 6.02, SE = 2.05; 95% CIs = 2.59 to 10.66; direct effects = 3.64, SE = 2.01, 95% CIs = −1.24 to 8.52).

## 4 DISCUSSION

Our previous study examined the close relationship between tau pathology and presynaptic density and between presynaptic density and postsynaptic density. However, the relationship between tau pathology and postsynaptic biomarkers has yet to be studied. Postsynaptic mGluR5 may play a vital role in the accumulation of hyperphosphorylated tau and triggering synaptic dysfunction via hyperphosphorylated tau.^44^, ^45^, ^46^ In this study, we found decreased mGluR5 availability in the hippocampus of AD patients using [^18^F]PSS232 PET. Hippocampal mGluR5 availability is associated with tau deposition in the hippocampus and most Braak stage regions. However, hippocampal tau deposition was not associated with mGluR5 in the neocortex. These results indicate a close correlation between tau and mGluR5, and the modulation of mGluR5 may protect synapses and further reduce tau phosphorylation, which could be used as a potential treatment for AD.

Our results are the first to indicate decreased mGluR5 availability in AD via PET and postmortem studies. These results are also in line with our previous results and other studies.^21^, ^22^, ^23^ mGluR5 availability reductions in the hippocampus may simply involve postsynaptic loss. However, controversial results have also been reported. As discussed previously, mGluR5 may be associated with the core pathology of AD at the early stage of the disease. However, mGluR5 is also expressed in glial cells and activated astrocytes. Therefore, in the late stage of this disease, mGluR5 expression is also increased with activated glia and astrocytes.

Tau is primarily an axonal protein that has been well‐documented to interact with and stabilize axonal microtubules. This interaction is crucial for maintaining the structural integrity and function of microtubules in neurons. Hyperphosphorylated tau can accumulate at pre‐ and postsynaptic terminals in the AD brain. In the postsynaptic terminal, phosphorylated tau has been argued to interact with Src kinase FYN and the complex formed by postsynaptic density 95 (PSD95) and *N*‐methyl‐d‐aspartate (NMDA) receptors, which may disrupt glutamate receptor signaling and transport and lead to decreased surface receptor expression. We observed a region‐to‐region correlation between tau deposition and mGluR5 only in the hippocampus. These findings indicate that hyperphosphorylated tau can participate in Aβ‐triggered or other pathways, leading to synaptic toxicity and loss in these regions. Phosphorylated tau promotes its interaction with FYN and facilitates FYN targeting of postsynaptic dendritic sites, leading to excitotoxic cascades.^48^ Therefore, hyperphosphorylated tau may impair presynapses and further impair synaptic transmission and postsynapses. These findings are consistent with those of a previous study of AD mouse models.^49^

Furthermore, we found that only hippocampal mGluR5 availability was associated with regional tau deposition, but that hippocampal tau deposition was not associated with regional mGluR5 availability. This finding is different from the closer association between tau and presynaptic SV2A. The transsynaptic propagation of tau pathology is important for synaptic plasticity, and aberrant synaptic activity has been shown to promote tau spreading.^50^ The transmission of phosphorylated tau from presynapses to postsynapses is the key step in the impairment of postsynaptic density. The aberrant mGluR5 is first altered in the hippocampus, and this pathogenic event of postsynaptic impairment may occur at a later stage than the tau pathology in the hippocampus. However, postsynaptic connections in the hippocampus occur in several other brain regions, and neurons in the hippocampus can receive phosphorylated tau from different brain regions through presynaptic and postsynaptic connections. Therefore, the correlation between tau and postsynaptic markers may not be synchronous. However, more evidence is needed to explain the asynchronous association between tau and postsynaptic markers.

Cognitive decline in AD also closely correlates with synaptic loss.^51^ In our study, mGluR5 in the hippocampus was positively correlated with MMSE, MoCA‐B, and episodic memory performance scores. Analysis of the mediation model suggested that the effects of mGluR5 on cognitive levels could be driven entirely by tau pathology. Hippocampal tau deposition may take part in the loss of postsynaptic mGluR5, which in turn leads to cognitive decline. A previous study examining the relationships among tau pathology, atrophy, and memory suggested that hippocampal atrophy mediates the effects of tau pathology on memory.^52^ Our results also indicated that the association between mGluR5 and cognition was dependent on tau. Taken together, these findings suggest that tau pathology in the hippocampus may lead to synaptic loss, which may accelerate hippocampal atrophy and ultimately lead to a decline in cognition.

The sex‐dependent contribution of mGluR5 to AD pathology was investigated by many groups; gender‐specific responses to AD therapies complicate the process of drug discovery. It has been found that mGluR5 availability by 3‐(6‐methylpyridin‐2‐ylethynyl)‐cyclohex‐2‐enone‐O‐11 C‐methyloxime ([^11^C]ABP688) BP_ND_ values is significantly higher in men compared to women.^53^ We also observed the similar pattern: hippocampal mGluR5 was slightly higher in men (SUVr: 1.949 vs 1.817; *p* = 0.09). Consistently, we found that hippocampal mGluR5 was related to increased hippocampal tau deposition and impaired MMSE and MoCA‐B scores only in women but not in men. Most studies show that women experience greater AD severity compared to men.^54^, ^55^, ^56^ Previous research has reported that women exhibit higher levels of tau deposition^57^, ^58^ and a faster rate of tau accumulation.^59^ In addition, cognitive decline progresses more rapidly in women than in men.^60^ Our complementary findings further highlight a higher prevalence of apolipoprotein E (*APOE*) ε4 allele carriage in women (18/23, *χ* ^2^ = 4.18, *p* = 0.041). Several studies indicate that *APOE* ε4 carriers in women exhibit elevated levels of CSF total tau and phosphorylated tau (p‐tau).^55^, ^61^ The combination of lower mGluR5 availability, greater tau burden, and associated cognitive decline may explain our finding of a significant correlation between mGluR5 and tau in the hippocampus exclusively in women.

Abd‐Elrahman et al. demonstrated that mGluR5 isolated from male mouse cortical and hippocampal tissues bound with high affinity to Aβ oligomers, whereas mGluR5 from female mice exhibited no such affinity, which meant that mGluR5 does not contribute to Aβ pathology in female mice.^62^ Furthermore, previous literature detected that mGluR5 depletion significantly impaired lipid metabolism, insulin sensitivity, and sympathetic output in women,^63^ underscoring the critical role of sex in mGluR5 function. Our finding of a hippocampal mGluR5‐tau correlation only in women highlights the complexity of mGluR5 pharmacology and the sex‐specific pathogenesis of AD.

This study has several limitations that need to be addressed. Because mGluR5 is overwhelmingly localized in postsynaptic terminals and sporadically localized in the presynaptic and nuclear membranes,^64^, ^65^, ^66^ it cannot fully demonstrate pathological changes in the postsynaptic membrane. Moreover, considering that this work was a single‐center study with a limited sample size, we should be cautious about drawing far‐reaching conclusions from these results. Finally, although our cross‐sectional findings suggest associations among mGluR5, tau pathology, and cognition, determining causal relationships requires longitudinal investigations, particularly in preclinical AD stages. Future studies should track the temporal sequence of mGluR5 changes and tau accumulation to establish whether mGluR5 modulation could effectively prevent tau‐mediated synapse loss.

## 5 CONCLUSION

We demonstrated that the decrease in hippocampal postsynaptic mGluR5 was reversed by the accumulation of pathologic tau in most cortical regions. Moreover, hippocampal mGluR5 availability is closely correlated with cognitive performance and is dependent on tau pathology in women. Our study suggests that mGluR5 could be a novel target for AD therapy by rescuing the spread of tau; however, it is necessary to reveal sex‐specific stratification in clinical trials.

## CONFLICT OF INTEREST STATEMENT

The authors report that they have no conflicts or competing interests. Author disclosures are available in the Supporting Information.

## CONSENT STATEMENT

This manuscript has been seen and approved by all authors. All authors declare no conflict of interest that may directly or indirectly influence the content of the manuscript submitted.

## Supporting information



Supporting Information

Supporting Information
