# Supplementary material for: Tau pathology is associated with postsynaptic metabotropic glutamate receptor 5 (mGluR5) in early Alzheimer's disease in a sex‐specific manner
Source: Alzheimers Dement. 2025 Feb 25;21(2):e70004. doi: 10.1002/alz.70004 (PMC11853735; doi:10.1002/alz.70004)
Supplement: Supplementary file 1 — Supporting Information [file ALZ-21-e70004-s002.docx]

**Supplemental materials**

**Tau pathology is associated with postsynaptic metabolic** **glutamate receptor 5 (mGluR5) in early Alzheimer’s disease in a sex-specific manner**

**Supplemental Table 1.** Postmortem human brain tissue

| **Group** | **n** | **Age (y)** | **Sex** | **APOE** **ε4 (0/1/2)** | **PMD (h)** | **Braak** | **Amyloid-β** |
| --- | --- | --- | --- | --- | --- | --- | --- |
| AD | 5 | 74.46 ± 7.50 | 4F/1M | 0/2/2 | 4.16 ± 0.55 | 5-6 | C |
| CU | 5 | 81.68 ± 8.05 | 3F/2M | 5/0/0 | 4.16 ± 1.99 | 1-3 | O-A |

Abbreviations: F: female; M: male; APOE: apolipoprotein E; PMD: postmortem delay.

**Supplemental Table 2. Information on the primary and 2^nd^ antibodies in detail**

| Reagent/Antibodies | Source | Identifier | Dilution |  |
| --- | --- | --- | --- | --- |
| Rabbit anti-mGluR5 | Abcam | ab76316 | 1:500 |  |
| Mouse anti-Abeta (6E10, IF) | Biolegend | 803004 | 1:1000 |  |
| Mouse phospho-Tau (Ser202, Thr205) monoclonal antibody (AT-8) | Invitrogen | MN1020 | 1:1000 | |
| AlphaTSA Multiplex IHC Kit | AlphaX Biotech | AXT37100031 | 1:100 |  |
| XTSA570 | AlphaX Biotech | AXT6410000 | 1:100 |  |
| XTSA620 | AlphaX Biotech | AXT6410000 | 1:100 |  |
| XTSA780 | AlphaX Biotech | AXT6110000 | 1:100 |  |
| Mouse purified anti-β-Amyloid, 17-24 monoclonal antibody (4G8, IHC) | 800701 | Biolegend | 1:4000 |  |
| Antibody diluent/block | AlphaX Biotech | AXT9310000 | / |  |
| Ethylenediaminetetraacetic acid | Zsbio | ZLI-9079 | 1:50 |  |
| Mounting medium | Phygene | PH0429 | / |  |
| DAPI | Invitrogen | P36970 | 1:10 |  |


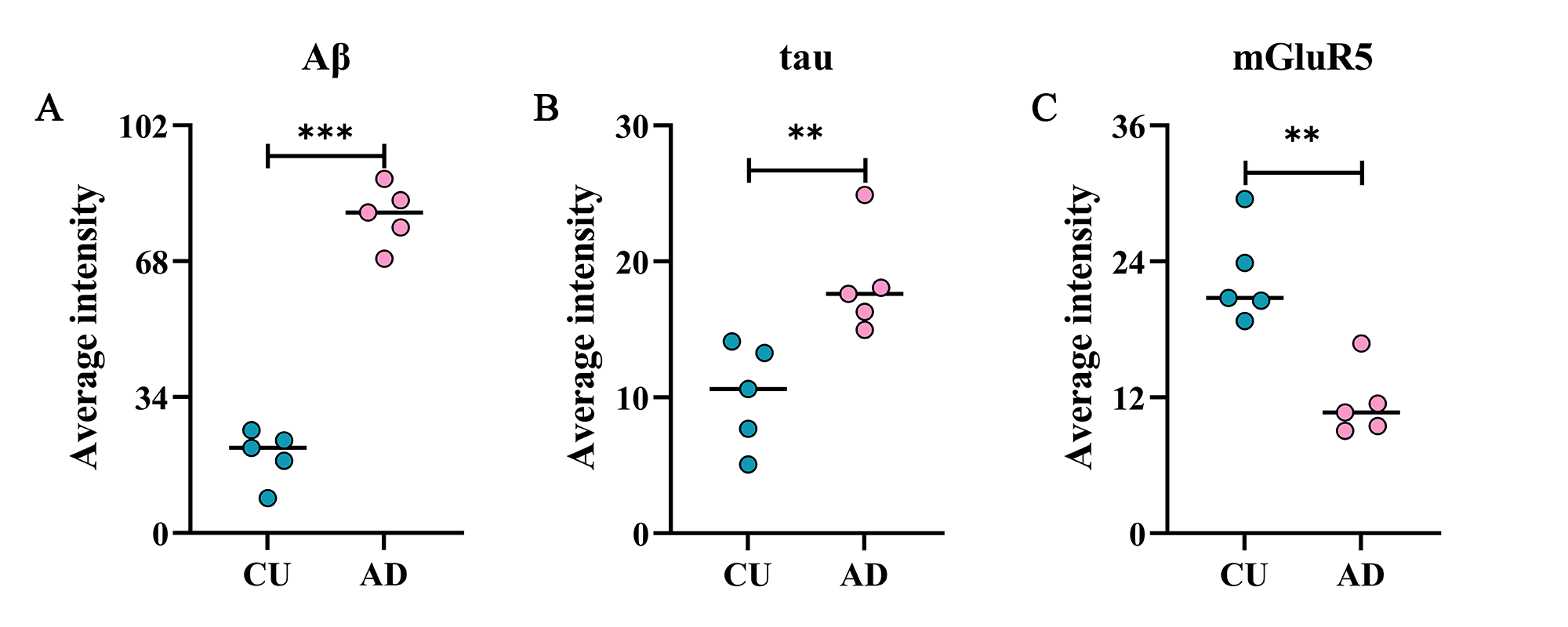


**Supplemental Figure 1.** Quantitative analysis of immunohistochemical staining of (A) Aβ, immunofluorescence staining of (B) tau and (C) mGluR5 in the postmortem hippocampus slices of AD and CU. The average % area of Aβ (4G8) and average fluorescence intensity of tau (AT-8) was higher in the hippocampus in AD (n = 5) than in CU individuals (n = 5), while the average fluorescence intensity of mGluR5 was lower in the hippocampus in AD (n = 5) than CU individuals (n = 5). **p < 0.01, ***p < 0.001.

**
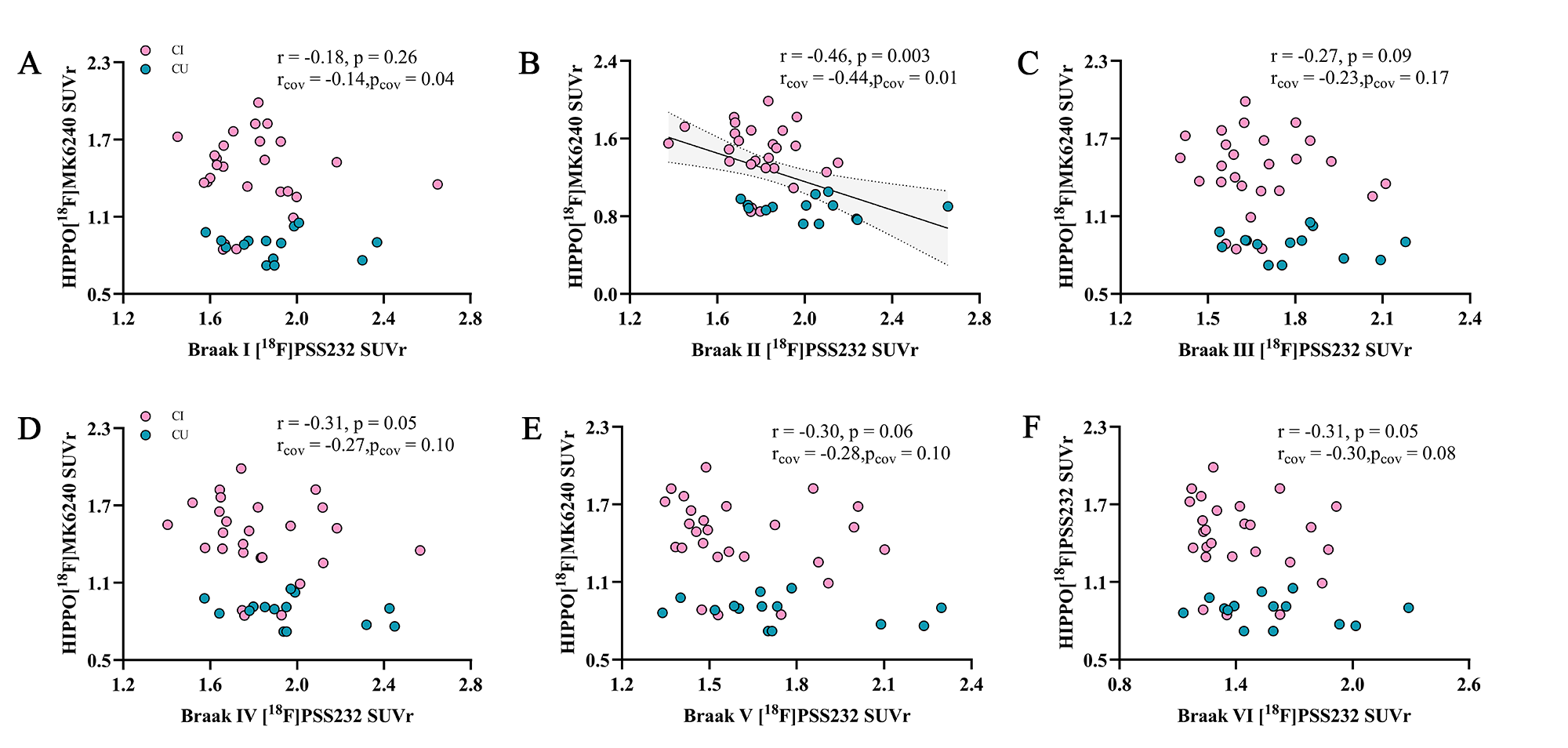
**

**Supplemental Figure 2.** Correlations between hippocampal [^18^F]MK6240 and [^18^F]PSS232 SUVr. There was no significant correlation between hippocampus tau deposition and [^18^F] PSS232 binding in Braak I region (A), Braak III region (C), Braak IV region (D), Braak V region (E) and Braak VI region (F) except Braak II region(B).


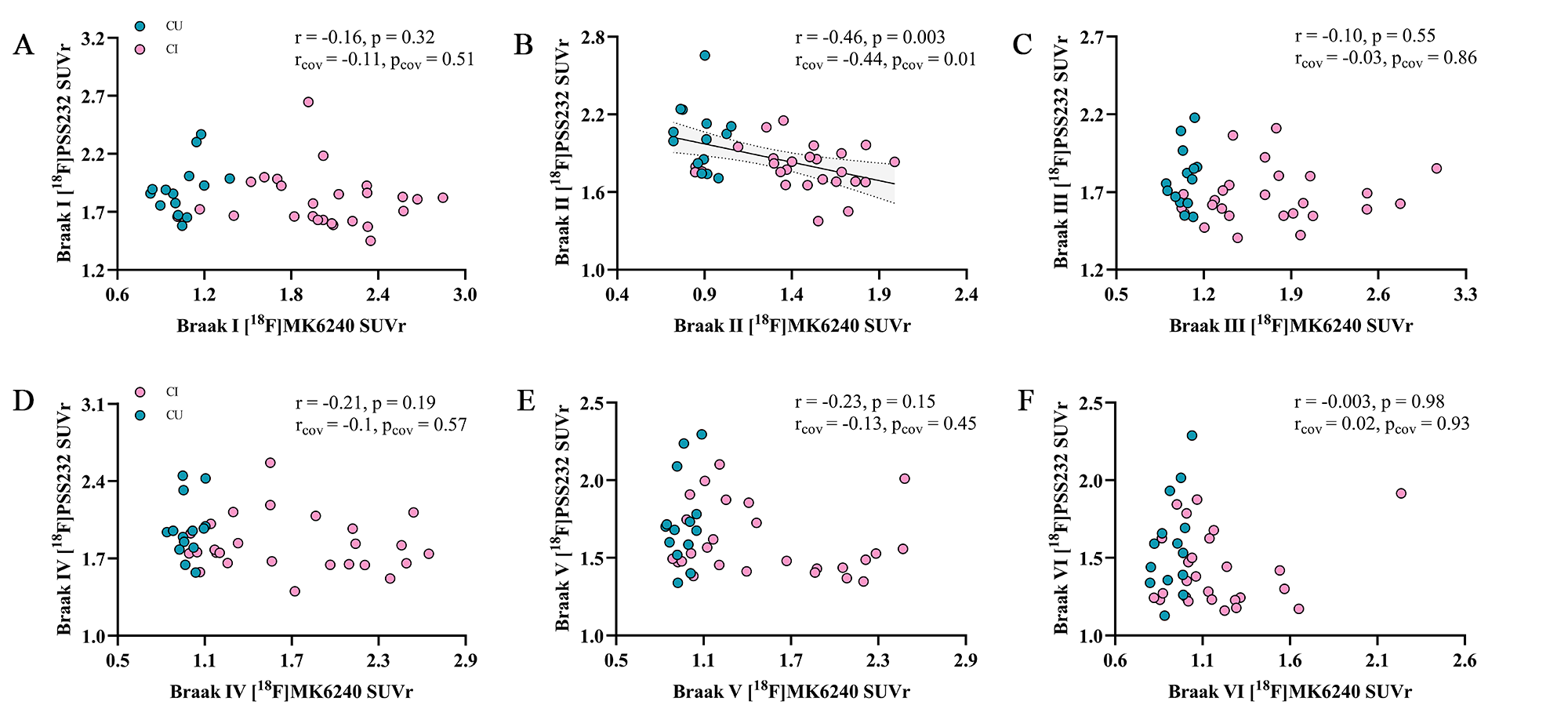


**Supplemental Figure 3.** Correlations between regional mGluR5 availability and tau deposition. There was no significant correlation between regional mGluR5 and regional tau tracer binding in Braak I region(A), Braak III region (C), Braak IV region (D), Braak V region (E) and Braak VI region (F) except Braak II region (B).
